# Evidential Uncertainty in Blood-Smear Image Classification: Mathematical Limits and Reliability Under Corruption

**DOI:** 10.3390/s26216957

**Published:** 2026-11-03

**Authors:** Umar Hasan, Muhammad Ali Martuza, Turki G. Alghamdi

**Affiliations:** 1 Department of Mathematics and Physics, School of Engineering and Physical Sciences, North South University, Dhaka 1229, Bangladesh; umar.hasan@northsouth.edu; 2 Department of Computer Engineering, College of Computer, Qassim University, Buraydah 51452, Saudi Arabia; 3 Department of Computer Engineering and Networks, College of Computer and Information Sciences, Jouf University, Sakaka 72388, Saudi Arabia; talghamdi@ju.edu.sa

**Keywords:** blood-smear image classification, medical image analysis, evidential deep learning, uncertainty quantification, calibration, image corruption, selective classification, subjective logic, noncoercivity, artificial intelligence

## Abstract

**Highlights:**

**What are the main findings?**

- Masked KL leaves target-evidence growth unpenalized along a noncoercive direction.
- Evidential uncertainty constrains confidence and imposes a Brier-loss floor.
- Five-seed blood-smear tests reveal corruption-dependent reliability trade-offs.

**What are the implications of the main findings?**

- Evidence strength alone cannot certify a prediction as safe to retain.
- Confidence-based selection and uncertainty-based error detection require separate evaluation.
- Clinical use requires patient-aware validation and independently chosen decision thresholds.

**Abstract:**

Reliable medical-image classification requires assessment of accuracy, calibration, and uncertainty under image degradation. We analyze the unit-prior Evidential Mean Squared Error (EMSE) objective with masked Kullback–Leibler (KL) regularization and evaluate four ResNet-18 classifiers for four-class blood-smear classification. We derive confidence–uncertainty bounds and a Brier-loss lower bound, and we prove that masked KL leaves a target-evidence direction along which the objective approaches an unattained zero infimum. Using five optimization seeds, we compare regularized evidential learning, its no-KL ablation, softmax, and 20-pass Monte Carlo dropout on 3242 external images across 12 corruption conditions. Mean clean accuracy exceeds 0.998 for all methods; softmax has the best mean clean Brier score, expected calibration error (ECE), area under the risk–coverage curve (AURC), and error-detection AUROC. Across corruptions, the no-KL ablation has the highest equal-weighted mean accuracy, while reliability rankings vary by corruption and metric. None of 390 paired comparisons is significant after Holm correction. These findings do not establish a general reliability benefit from masked KL and clarify the distinct roles of predictive confidence and evidential uncertainty.

## 1. Introduction

Reliable neural networks for real-world data must maintain useful predictive behavior beyond the conditions under which their parameters were optimized. Accuracy alone is insufficient: reliability also depends on calibration, uncertainty ranking, optimization stability, and transparent limits on the guarantees supported by the training objective [1]. Representation choices can further affect reliability; recent work has linked representation design to safety-critical tabular prediction [2] and has shown that latent-space entanglement and token fragmentation can produce cross-lingual reliability failures [3]. Standard deep-learning classifiers typically use a softmax activation to project unbounded logits onto the categorical probability simplex. Although computationally efficient, this deterministic mapping does not explicitly distinguish aleatoric uncertainty, which arises from intrinsic data ambiguity, from epistemic uncertainty, which represents model ignorance [4]. Consequently, softmax-based networks can be poorly calibrated [5] and can produce highly confident predictions far from the training distribution [6,7,8,9].

Evidential deep learning (EDL) provides a higher-order uncertainty representation through the principles of subjective logic [10,11]. Instead of producing only point estimates of class probabilities, an evidential network predicts the concentration parameters of a Dirichlet distribution [12,13]. This construction represents classification as an evidence-acquisition process and supplies a subjective uncertainty mass inversely related to total evidence [14]. We examine this mass as a candidate epistemic uncertainty signal, rather than assuming that its value directly establishes predictive reliability. Unlike Monte Carlo dropout [15] and deep ensembles [16], the resulting uncertainty estimate requires only one deterministic forward pass.

Despite the appeal of this construction, evidential loss manifolds introduce nontrivial optimization challenges. The empirical-risk term, commonly implemented as an Evidential Mean Squared Error (EMSE), may encourage increasingly concentrated Dirichlet densities and thereby contribute to overconfident predictions [17]. A Kullback–Leibler (KL) divergence penalty is often introduced to suppress evidence associated with incorrect classes. However, if this penalty is applied too strongly during early training, it can drive the network toward an uninformative uniform-evidence state and impair convergence [18].

The mathematical difficulty arises because an evidential classifier must learn two coupled quantities: a direction in the probability simplex and a radial evidence scale. The direction determines the predictive mean, while the scale controls the concentration of the Dirichlet density and the subjective uncertainty mass. The EMSE is derived from an expected quadratic scoring rule related to the Brier score [19,20]; it rewards accurate predictive means and small posterior variance but does not, by itself, assign a semantic penalty to evidence supporting an incorrect class. The masked KL term supplies this missing mechanism by comparing the non-target concentration vector with a unit Dirichlet reference [21]. Understanding the interaction of these terms is essential because accuracy, calibration, and epistemic uncertainty are distinct statistical properties [22,23].

This article investigates evidential uncertainty in four-class blood-smear image classification. The empirical question is whether uncertainty estimates remain useful for identifying classification errors as image quality changes and how this behavior relates to probability calibration and confidence-ranked prediction retention. We explicitly distinguish between the well-established baseline properties of the EMSE formulation and our novel analytical contribution regarding the noncoercivity of the masked EMSE-KL objective along the target ray. We evaluate available digital images from a primary corpus and a separate external repository, with blur, brightness, stain, and noise transformations providing controlled stress conditions. The study concerns image-level model behavior: it does not evaluate image acquisition, patient-level diagnosis, or subsequent clinical decisions. This distinction allows the medical-image application to inform the reliability analysis without treating retrospective image-classification results as evidence of clinical readiness.

The mathematical results concern the stated Dirichlet parameterization, unit prior, and masked objective; they do not depend on blood-smear morphology and are intended to characterize the model formulation rather than a disease-specific mechanism. The blood-smear experiment is a bounded application case study: microscopy makes focus, illumination, staining, and sensor-noise perturbations interpretable, but this setting alone cannot establish that the empirical findings generalize across diseases, datasets, or architectures.

The central mathematical question is whether masked KL resolves the radial scale degeneracy of EMSE. We show that it does not: masking makes the regularizer independent of target evidence, so the complete objective has an unattained zero infimum along a pure target-evidence ray. A confidence–uncertainty envelope and an uncertainty-dependent Brier-loss floor further constrain how evidence can inform prediction selection. A five-seed external stress test compares regularized EDL, EDL without KL, softmax, and Monte Carlo dropout under four corruption families and three severity levels. Confidence-ranked selective risk and uncertainty-ranked error detection are evaluated separately. The contribution combines analysis of the evidential representation with a comparative blood-smear corruption study; it is not a new backbone, an operational referral policy, or a guarantee of robustness.

The study is guided by the following theoretical and empirical hypotheses:

**Hypothesis** **1.** *The masked KL divergence has a unique minimum at the unit Dirichlet distribution and therefore contracts unsupported non-target evidence toward zero.*

**Hypothesis** **2.** *The masked KL term improves non-target evidence control relative to an otherwise identical unregularized evidential model, particularly for uncertainty-based error ranking under corruption.*

**Hypothesis** **3.** *The single-pass evidential model exhibits reliability trade-offs that differ from softmax and Monte Carlo dropout as corruption severity increases.*

The key contributions of this work are as follows:

- We formulate the complete logit–evidence–opinion mapping and show explicitly that the projected subjective probability equals the mean of the associated Dirichlet distribution.
- We derive global finite bounds and an exact expected-risk decomposition for the EMSE, together with an uncertainty-dependent lower bound on the Brier component and an exact confidence–uncertainty envelope.
- We characterize evidence-ray limits and prove that the complete EMSE–masked-KL objective is noncoercive, with an unattained zero infimum along the target-evidence direction.
- We derive the gradient and local Hessian of the masked Dirichlet KL penalty and clarify its precise scope: it contracts non-target evidence but is mathematically blind to target-evidence inflation.
- We evaluate how image corruptions affect blood-smear classification across five optimization seeds, comparing regularized EDL, EDL without KL, softmax, and Monte Carlo dropout through calibration, confidence-ranked selective risk, uncertainty-based error detection, recorded inference cost, and a paired seed-wise comparison.

The remainder of the article is organized as follows. Section 2 reviews uncertainty quantification, subjective logic, calibration, and evidential optimization. Section 3 defines the image-level evaluation scope and prediction-selection protocol, develops the mathematical results, and documents the comparative stress test. Section 4 reports the analytical and empirical findings. Section 5 interprets their implications for medical-image classification and the validation required before clinical use, and Section 6 concludes the article.

## 2. Related Work

Research on reliable prediction spans Bayesian inference, ensemble learning, post hoc calibration, distribution-shift detection, and higher-order probability models. These families differ in how they represent uncertainty, whether inference requires repeated model evaluations, and which aspects of their objectives admit mathematical analysis. Table 1 summarizes representative methods and positions the present study as an analysis of evidential loss geometry and masked regularization, complemented by external image-corruption evaluation.

More broadly, recent mathematical AI research has examined complementary aspects of reliability and robustness, including activation design against adversarial examples [24], theoretically validated representation and clustering methods for evolving networks [25], multimodal augmentation for knowledge-graph completion [26], and inference-time mitigation of memorization in generative models [27]. These studies illustrate the diversity of mathematical mechanisms used to address reliability-related challenges; the present work focuses specifically on predictive uncertainty, calibration, and evidential loss geometry.

**Table 1:** Representative uncertainty-quantification and calibration approaches related to the present work.

Reference	Representation	Principal Contribution	Limitation Relative to This Study
Gal and Ghahramani [15]	Approximate posterior induced by dropout	Interprets stochastic dropout inference as approximate Bayesian inference.	Requires multiple stochastic forward passes for predictive moments.
Lakshminarayanan et al. [16]	Ensemble of predictive distributions	Provides strong uncertainty and calibration under distribution shift.	Multiplies training, storage, and inference cost.
Malinin and Gales [28]	Dirichlet prior network	Separates distributional uncertainty from data uncertainty using a distribution over categorical distributions.	Requires explicit in-domain and out-of-domain training objectives.
Sensoy et al. [12]	Subjective opinion/Dirichlet density	Introduces single-pass evidential classification and the EMSE–KL objective.	Provides limited analysis of loss bounds and optimization geometry.
Guo et al. [5]	Temperature-scaled probabilities	Establishes modern neural-network miscalibration and an effective post hoc baseline.	Does not represent epistemic evidence or higher-order uncertainty.
Kull et al. [29]	Dirichlet calibration map	Generalizes post hoc calibration to native multiclass transformations.	Calibrates point probabilities after training rather than learning evidence.
Ovadia et al. [22]	Shift-based empirical benchmark	Demonstrates that in-domain calibration need not transfer under dataset shift.	Primarily empirical; it does not characterize evidential loss geometry.
Franzen and Pourkamali-Anaraki [18]	Annealed Dirichlet classifier	Shows reductions in confident errors and emphasizes stable KL annealing.	Does not derive the EMSE bounds and local KL curvature developed here.

### 2.1. Probabilistic Uncertainty Quantification in Deep Learning

The need for trustworthy artificial intelligence has motivated extensive research into uncertainty quantification [1,30]. Bayesian neural networks and deep ensembles approximate predictive uncertainty through posterior inference or repeated model evaluation [15,16,31]. Although effective, these approaches can impose substantial computational costs during training or inference [9]. Prior Networks directly predict a distribution over categorical distributions and explicitly represent distributional uncertainty [28]. Evidential models adopt a related Dirichlet representation but learn evidence through a deterministic network in a single forward pass, thereby offering a computationally efficient uncertainty mechanism. In medical imaging, ordinal conformal prediction has likewise shown that approximately nominal internal coverage can deteriorate and become class-specific during external evaluation, even when prediction sets are constrained to clinically coherent adjacent grades [32].

### 2.2. Mathematical Foundations of Evidential Deep Learning

The integration of subjective logic into deep learning interprets neural outputs as evidence assigned to mutually exclusive hypotheses [10,11,12,13]. The opinion consists of classwise belief masses, an uncertainty mass, and a base rate; under a unit Dirichlet prior, its projected probability coincides with the Dirichlet mean. Recent work has generalized this construction. Flexible Evidential Deep Learning extends the standard Dirichlet formulation to improve expressiveness under noisy conditions [33], whereas Re-EDL questions several conventional regularization terms and analyzes how they affect the probabilistic interpretation of the predicted Dirichlet density [17]. The present work complements these studies by focusing on the bounds, radial degeneracy, KL curvature, and convergence properties of the evidential loss.

### 2.3. Optimization, Calibration, and Loss Manifolds

Optimizing evidential concentration parameters raises distinct questions concerning loss geometry and gradient flow. The original EMSE combines a proper quadratic component with a Dirichlet variance term [12,19,20]. Tangential Gradient Descent has been proposed to improve angular class separation and uncertainty estimation as the number of classes increases [14]. Annealed KL penalties can reduce high-confidence errors while avoiding early training collapse [18]. Related approaches combine topological risk analysis with Dirichlet uncertainty [34] or introduce class-balanced losses and learned priors for skewed data [35,36]. Because the implementation uses Adam, its empirical convergence should also be distinguished from deterministic gradient-descent theory; convergence failures and corrected adaptive methods are discussed in [37,38].

### 2.4. Applications of Evidential Optimization

Evidential uncertainty has been incorporated into federated learning [39], out-of-distribution detection [40], medical image segmentation [41], multimodal data fusion [42,43], and uncertainty-aware image retrieval [44]. Related medical-imaging research has also demonstrated that parameter-efficient low-rank attention adaptation can retain strong cross-dataset segmentation performance under domain shift [45], illustrating the broader importance of efficient and reliable model adaptation. Combining evidential networks with uncertainty-aware training has also been shown to improve calibration in complex artifact-detection tasks [46]. Nevertheless, calibration estimates are sensitive to binning and finite-sample bias [47,48,49]; therefore, the present analysis reports the Brier score together with ECE, reliability curves, discrimination metrics, and uncertainty distributions rather than treating a single calibration number as definitive.

For the same four-class ALL setting considered here, NeurALLNet combines spiking computation and channel attention to obtain a compact classifier and reports evaluation on the same 3242-image external cohort [50]. That study also identifies the absence of patient-level metadata and the common geographic origin of the two public repositories as limitations of subject-independent and cross-regional interpretation.

## 3. Methods

**Figure 1:**
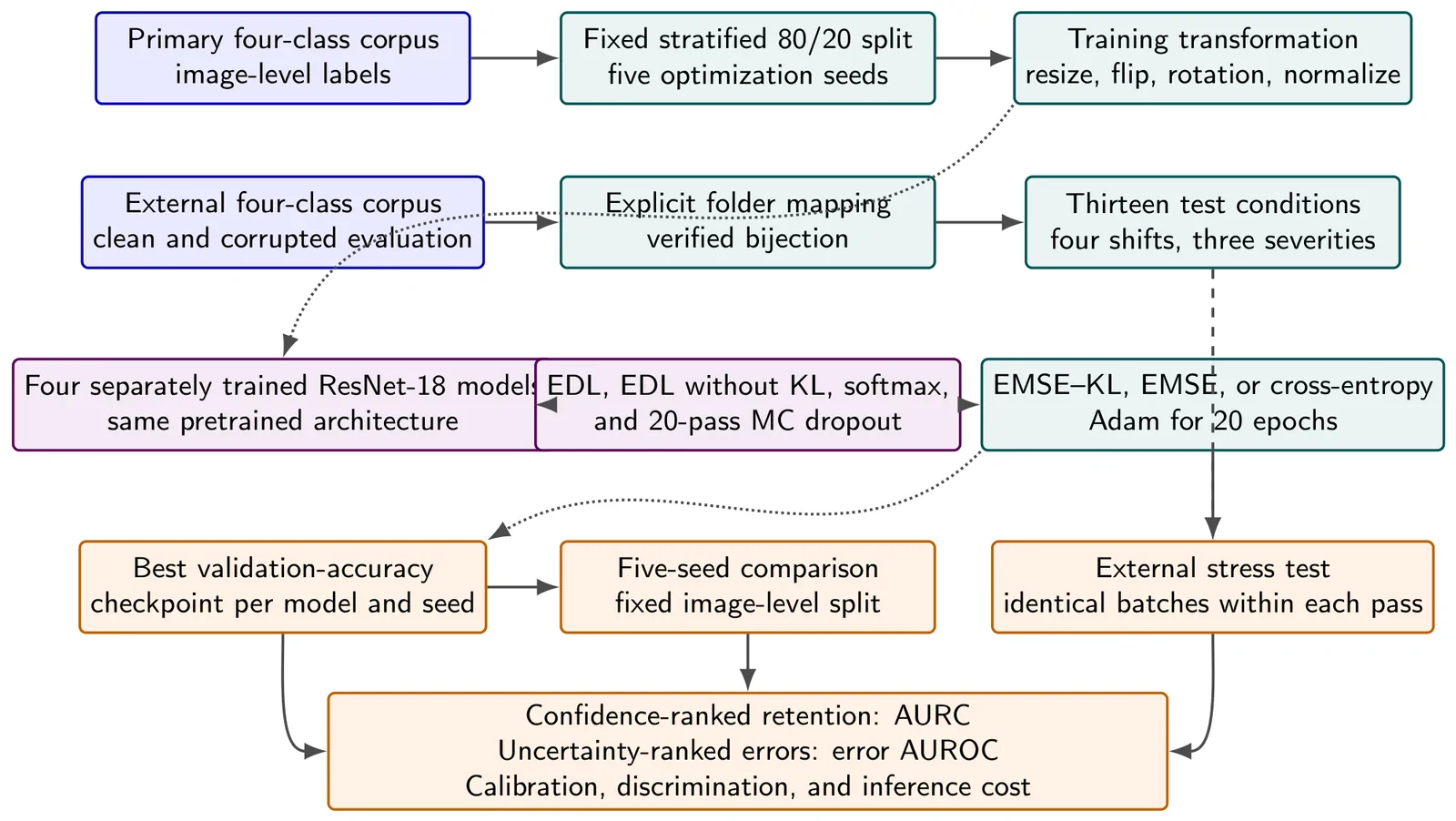
Experimental workflow connecting model development and the external input environment to prediction-selection and reliability evaluation. The same realized external batch is supplied to all models within a pass. Confidence-based AURC and uncertainty-ranked error detection are distinct retrospective assessments; neither constitutes validation of a clinical referral workflow.

This section presents the mathematical analysis and blood-smear image evaluation protocol. We define the image-level scope and evidential learning problem, formalize confidence-based retention, and analyze the EMSE and KL terms, including conditional post-annealing convergence. The remaining subsections document the datasets, preprocessing, ResNet-18 mapping, optimization procedure, external-validation protocol, and evaluation metrics. The complete experiment code is publicly available at https://github.com/umarbhasan/conditional-convergence (accessed on 26 October 2026). Figure 1 summarizes the experimental workflow; prediction selection is specified in Section 3.3.

### 3.1. Image-Level Evaluation Scope

The evaluated pipeline comprises blood-smear image preprocessing, a trained classifier, probability and uncertainty outputs, and retrospective prediction selection. A separate image repository and controlled blur, brightness, stain, and noise transformations provide external evaluation conditions. Their effects are assessed through prediction errors, calibration, error ordering, retained risk, and inference cost. Training and checkpoint selection form the development stage, whereas the external collection is reserved for evaluation. These image transformations are stress tests, not a validation of specific microscope hardware or acquisition protocols.

At the output boundary, abstention means excluding a prediction from the retained set used to calculate selective risk. It does not mean that the corresponding patient received a referral or that a clinician corrected the error. The study does not model laboratory acquisition, staffing constraints, downstream diagnostic costs, or feedback from human decisions. It therefore evaluates a bounded digital decision-support component and its selection interface, rather than an end-to-end healthcare service.

### 3.2. Problem Formulation

Let $X\subseteq R^{H\times W\times 3}$ denote the image space and let Y={1,…,K} denote the set of mutually exclusive classes. The training sample is

(1) $$D_{\mathrm{tr}}={\left\{\left(x_{i},y_{i}\right)\right\}}_{i=1}^{N_{\mathrm{tr}}},\;x_{i}\in X,\;y_{i}\in Y,$$

and $e_{y_{i}}\in {\left\{0,1\right\}}^{K}$ denotes the one-hot vector associated with $y_{i}$. A neural mapping $f_{\Theta }:X\to R^{K}$ produces logits $z=f_{\Theta }\left(x\right)$. Rather than applying softmax, the evidential model constructs a Dirichlet density

(2) $$q_{\Theta }\left(\pi |x\right)=\mathrm{Dir}\;\left(\pi |\alpha_{\Theta }\left(x\right)\right),\;\pi \in \Delta^{K-1},$$

where $\Delta^{K-1}=\left\{\pi \in R_{+}^{K}:\sum_{k}\pi_{k}=1\right\}$ is the categorical probability simplex. The model must simultaneously learn (i) a predictive direction, represented by the Dirichlet mean, and (ii) an evidence scale, represented by the strength *S*. The decision and uncertainty functions are

(3) $${\hat{y}}_{\Theta }\left(x\right)=\arg\max_{k}\frac{\alpha_{\Theta ,k}\left(x\right)}{S_{\Theta }\left(x\right)},\;u_{\Theta }\left(x\right)=\frac{K}{S_{\Theta }\left(x\right)}.$$

**Problem** **1** (Regularized Evidential Risk Minimization)**.** *Determine parameters $\Theta^{★}$ that minimize the empirical objective*

(4) $$\Theta^{★}\in \arg\min_{\Theta }\frac{1}{N_{\mathrm{tr}}}\sum_{i=1}^{N_{\mathrm{tr}}}\left[L_{\mathrm{EMSE}}\;\left(\alpha_{\Theta }\left(x_{i}\right),e_{y_{i}}\right)+\lambda_{t}L_{\mathrm{KL}}\;\left({\tilde{\alpha }}_{\Theta }\left(x_{i}\right),e_{y_{i}}\right)\right],$$

*while preserving high classification accuracy and ensuring that unsupported non-target evidence is penalized. The time-varying coefficient $\lambda_{t}$ must be chosen so that evidence discrimination is learned before the regularizer reaches full strength.*

The formulation differs from ordinary probabilistic classification because two networks can have the same mean probability vector but different strengths. They therefore make the same class decision while expressing different subjective uncertainty masses. This non-identifiability at the level of point probabilities motivates analysis of the radial behavior of the loss; the uncertainty mass is not itself an observed error probability.

### 3.3. Confidence-Based Prediction Selection

Let $c_{\Theta }\left(x\right)=\max_{k}p_{\Theta ,k}\left(x\right)$ denote top-1 confidence. A threshold τ∈[0,1] defines a retention indicator $a_{\tau }\left(x\right)=1\left\{c_{\Theta }\left(x\right)\geq \tau \right\}$: the system retains ${\hat{y}}_{\Theta }\left(x\right)$ when $a_{\tau }\left(x\right)=1$ and otherwise abstains. On an evaluation set of *n* images, empirical coverage and selective risk are

(5) $$\hat{C}\left(\tau \right)=\frac{1}{n}\sum_{i=1}^{n}a_{\tau }\left(x_{i}\right),\;\hat{R}\left(\tau \right)=\frac{\sum_{i=1}^{n}a_{\tau }\left(x_{i}\right)1\left\{{\hat{y}}_{\Theta }\left(x_{i}\right)\neq y_{i}\right\}}{\sum_{i=1}^{n}a_{\tau }\left(x_{i}\right)}.$$

Selective risk is defined only for nonempty retained sets. Risk–coverage curves can be computed by evaluating successive prefixes after sorting predictions by decreasing confidence; tied scores may be split by the sorting order. This is a retrospective assessment across coverage levels, not a threshold selected on independent validation data and then tested as a fixed deployment rule. AURC summarizes this confidence-based ordering; the results below report seed-level AURC rather than per-image curves.

Error-detection AUROC instead evaluates the model-specific uncertainty score: evidential mass *u*, softmax predictive entropy, or MC-dropout mutual information. These scores need not induce the same ranking as top-1 confidence. We therefore distinguish confidence-based selective performance from uncertainty-based error detection throughout the evaluation. An operational uncertainty-threshold policy and its downstream consequences are not tested here.

### 3.4. Mathematical Formulation of Evidential Deep Learning

Let $Z=\left(z_{1},\ldots ,z_{K}\right)\in R^{K}$ denote the continuous output logits of a neural network for a classification problem with *K* mutually exclusive classes.

**Definition** **1** (Evidence Mapping and Dirichlet Parameterization)**.** *The nonnegative evidence vector $E=(e_{1},\ldots ,e_{K})$ is obtained using the softplus mapping*

(6) $$e_{k}=\log\;\left(1+\exp(z_{k})\right),\;k=1,\ldots ,K.$$

*The corresponding Dirichlet concentration parameters are*

(7) $$\alpha_{k}=e_{k}+1.$$

**Definition** **2** (Subjective Probability and Uncertainty)**.** *The Dirichlet strength and expected class probability are*

(8) $$S=\sum_{k=1}^{K}\alpha_{k},\;p_{k}=E\left[P_{k}\right]=\frac{\alpha_{k}}{S}.$$

*Under the standard unit-prior subjective-logic correspondence, the subjective uncertainty mass, interpreted as a candidate epistemic signal, is*

(9) $$u=\frac{K}{S}.$$

*The classwise belief masses and uniform base rates are*

(10) $$b_{k}=\frac{e_{k}}{S}=\frac{\alpha_{k}-1}{S},\;a_{k}=\frac{1}{K}.$$

*They satisfy the subjective-opinion conservation law and projection rule*

(11) $$\sum_{k=1}^{K}b_{k}+u=1,\;b_{k}+a_{k}u=\frac{\alpha_{k}-1}{S}+\frac{1}{S}=\frac{\alpha_{k}}{S}=p_{k}.$$

**Proposition** **1** (Confidence–Uncertainty Coupling)**.** *For the unit-prior parameterization, every class probability satisfies*

(12) $$\frac{u}{K}\leq p_{k}\leq 1-\frac{K-1}{K}u.$$

*Consequently, if $c=\max_{k}p_{k}$ denotes top-1 confidence, then*

(13) $$\frac{1}{K}\leq c\leq 1-\frac{K-1}{K}u,\;u\leq \frac{K(1-c)}{K-1}.$$

*Proof provided in the Supplementary Materials.*

**Proposition** **2** (Uncertainty-Induced Brier Floor)**.** *Let $y=e_{t}$ be a one-hot target. For any unit-prior subjective opinion with uncertainty u, the squared error of the projected mean satisfies*

(14) $${∥y-p∥}_{2}^{2}\geq \frac{K-1}{K}u^{2}.$$

*Equality holds when all committed belief is assigned to the target class, namely $b_{t}=1-u$ and $b_{j}=0$ for j≠t.*

*Proof provided in the Supplementary Materials.*

#### 3.4.1. Expected Quadratic Risk and EMSE Decomposition

For a one-hot target vector $y=(y_{1},\ldots ,y_{K})$, the EMSE combines the squared error of the Dirichlet mean with the predictive variance:

(15) $$L_{\mathrm{EMSE}}\left(\alpha ,y\right)=\sum_{k=1}^{K}\left[{\left(y_{k}-\frac{\alpha_{k}}{S}\right)}^{2}+\frac{\alpha_{k}\left(S-\alpha_{k}\right)}{S^{2}\left(S+1\right)}\right].$$

Equation (15) follows from integrating the quadratic classification loss over the Dirichlet density. If P∼Dir(α), then

(16) $$\begin{array}{cc} E\;\left[{∥y-P∥}_{2}^{2}\right] & ={∥y-E\left[P\right]∥}_{2}^{2}+\mathrm{tr}\;\left(\mathrm{Cov}(P)\right)\\ & =\sum_{k=1}^{K}{\left(y_{k}-p_{k}\right)}^{2}+\sum_{k=1}^{K}\frac{\alpha_{k}\left(S-\alpha_{k}\right)}{S^{2}\left(S+1\right)}. \end{array}$$

The first term is a multiclass Brier loss evaluated at the Dirichlet mean, and the second is the trace of the Dirichlet covariance. This identity makes clear that the EMSE penalizes both mean error and posterior dispersion, but does not directly encode whether a large concentration is attached to a target or non-target class.

**Proposition** **3** (Bounds of the Evidential Mean Squared Error)**.** *For $\alpha_{k}\geq 1$ and a one-hot target y, the EMSE satisfies*

(17) $$0\leq L_{\mathrm{EMSE}}\left(\alpha ,y\right)\leq 2+\frac{K-1}{K(K+1)}.$$

*Proof provided in the Supplementary Materials.*

**Proposition** **4** (Evidence-Ray Limit and Scale Degeneracy)**.** *Let $r\in R_{+}^{K}$ with $R=\sum_{k}r_{k}>0$, and define an evidence ray by*

(18) $$\alpha_{k}\left(c\right)=1+cr_{k},\;c\geq 0.$$

*Then*

(19) $$\lim_{c\to \infty }p_{k}\left(c\right)=\frac{r_{k}}{R},\;\lim_{c\to \infty }u\left(c\right)=0,\;\lim_{c\to \infty }L_{\mathrm{EMSE}}\left(\alpha \left(c\right),y\right)={∥y-\frac{r}{R}∥}_{2}^{2}.$$

*Proof provided in the Supplementary Materials.*

**Corollary** **1** (Unregularized Evidence Inflation)**.** *If the evidence direction satisfies r/R=y, then the EMSE converges to zero as c→∞ while the subjective uncertainty also converges to zero. Hence, the unregularized EMSE can reward arbitrarily large evidence on correctly oriented samples and does not provide a finite preferred evidence scale.*

#### 3.4.2. Masked KL Regularization and Local Geometry

To penalize evidence assigned to non-target classes, the target-class concentration is masked as

(20) $${\tilde{\alpha }}_{k}=y_{k}+\left(1-y_{k}\right)\alpha_{k}.$$

The divergence from the noninformative Dirichlet distribution Dir(1) is

(21) $$\begin{array}{cc} L_{\mathrm{KL}}\left(\tilde{\alpha }\right)= & \log\Gamma \;\left(\sum_{k=1}^{K}{\tilde{\alpha }}_{k}\right)-\sum_{k=1}^{K}\log\Gamma \left({\tilde{\alpha }}_{k}\right)-\log\Gamma \left(K\right)\\ & +\sum_{k=1}^{K}\left({\tilde{\alpha }}_{k}-1\right)\left[\psi \left({\tilde{\alpha }}_{k}\right)-\psi \;\left(\sum_{j=1}^{K}{\tilde{\alpha }}_{j}\right)\right], \end{array}$$

where Γ(·) is the Gamma function and ψ(·) is the digamma function.

**Theorem** **1** (Regularization of Non-Target Evidence)**.** *For the masked concentration vector in Equation (20), $L_{\mathrm{KL}}\geq 0$, with equality if and only if $\tilde{\alpha }=1$. Consequently, the unique minimum of the KL term satisfies $e_{k}=0$ for every non-target class k.*

**Proof.**  The quantity in Equation (21) is the KL divergence $D_{\mathrm{KL}}\left[\mathrm{Dir}\left(\tilde{\alpha }\right)∥\mathrm{Dir}\left(1\right)\right]$. By the nonnegativity and identity properties of KL divergence, it is zero exactly when the two Dirichlet densities are equal, which requires ${\tilde{\alpha }}_{k}=1$ for all *k*. For a non-target class, $y_{k}=0$ and hence ${\tilde{\alpha }}_{k}=\alpha_{k}=e_{k}+1$. Therefore, ${\tilde{\alpha }}_{k}=1$ implies $e_{k}=0$.    □

The first-order geometry of the regularizer can be written in closed form. Let $A=\sum_{k}{\tilde{\alpha }}_{k}$ and let $\psi_{1}$ denote the trigamma function. Differentiating Equation (21) gives

(22) $$\frac{\partial L_{\mathrm{KL}}}{\partial {\tilde{\alpha }}_{l}}=\left({\tilde{\alpha }}_{l}-1\right)\psi_{1}\left({\tilde{\alpha }}_{l}\right)-\left(A-K\right)\psi_{1}\left(A\right).$$

At the uniform point $\tilde{\alpha }=1$, the gradient vanishes. The Hessian at that point is

(23) $$H_{\mathrm{KL}}\left(1\right)=\psi_{1}\left(1\right)I_{K}-\psi_{1}\left(K\right)11^{T}.$$

Its eigenvalues are $\psi_{1}\left(1\right)$ on the (K−1)-dimensional subspace orthogonal to 1 and $\psi_{1}\left(1\right)-K\psi_{1}\left(K\right)$ in the radial direction. Both are positive for K≥2, so the uniform Dirichlet is a strict local minimum as well as the unique global minimum. In the masked loss, the target coordinate is fixed at one; restricting Equation (23) to the non-target coordinates preserves positive definiteness. This curvature explains why the regularizer contracts small non-target evidence perturbations toward zero.

**Theorem** **2** (Noncoercivity of the Regularized Objective)**.** *Let $y=e_{t}$ and consider the pure target-evidence ray*

(24) $$\alpha \left(c\right)=1+ce_{t},\;c\geq 0.$$

*On the closed concentration domain $\alpha_{k}\geq 1$, and for every KL weight λ≥0, the per-sample regularized objective*

(25) $$J_{\lambda }\left(\alpha ,y\right)=L_{\mathrm{EMSE}}\left(\alpha ,y\right)+\lambda L_{\mathrm{KL}}\left(\tilde{\alpha }\right)$$

*satisfies*

(26) $$J_{\lambda }\left(\alpha \left(c\right),y\right)=\frac{K(K-1)}{{\left(K+c\right)}^{2}}+\frac{\left(K-1)(K+2c\right)}{{\left(K+c\right)}^{2}\left(K+c+1\right)}>0$$

*for finite c, while $J_{\lambda }\left(\alpha \left(c\right),y\right)\to 0$ as c→∞. Hence the objective is noncoercive in concentration space and its infimum along this ray is zero but is not attained at finite concentration.*

**Proof.**  Masking replaces the target coordinate by one and leaves every non-target coordinate equal to one; therefore $\tilde{\alpha }\left(c\right)=1$ and the KL term is identically zero for all *c*. On the ray, S=K+c, $p_{t}=\left(1+c\right)/S$, and $p_{j}=1/S$ for j≠t. The squared mean error is $K\left(K-1\right)/S^{2}$. The summed Dirichlet variance is

(27) $$\frac{1-\sum_{k}p_{k}^{2}}{S+1}=\frac{\left(K-1)(K+2c\right)}{S^{2}\left(S+1\right)}.$$

Both terms are positive at finite *c* and converge to zero, proving Equation (26) and noncoercivity.    □

**Remark** **1** (Scope of Masked Regularization)**.** *The unique minimum and positive curvature of the KL term concern the masked concentration vector, not the unmasked target coordinate. The regularizer therefore resolves unsupported non-target evidence locally and globally, but it cannot select a finite target-evidence scale. This limitation remains for every annealing coefficient and is distinct from whether a numerical optimizer reaches a stationary point in network-parameter space.*

*For finite softplus logits, every concentration is strictly greater than one, so the boundary ray in Equation (24) is approached rather than attained. Taking the target logit to +∞ and the non-target logits to −∞ approximates the same sequence arbitrarily closely; the noncoercive infimum therefore persists for the implemented parameterization.*

#### 3.4.3. Annealed Objective and Conditional Convergence

The epoch-dependent training objective is

(28) $$L_{t}=L_{\mathrm{EMSE}}+\lambda_{t}L_{\mathrm{KL}},\;\lambda_{t}=\min\;\left(1,\frac{t}{t_{\mathrm{anneal}}}\right).$$

In the implementation, epochs are zero-indexed: t=0,…,19 and $t_{\mathrm{anneal}}=10$. Consequently, the first displayed training epoch uses $\lambda_{0}=0$, the coefficient increases by 0.1 per epoch, and full KL strength is first reached at zero-indexed epoch 10 (the eleventh displayed epoch). The schedule initially prioritizes class discrimination and progressively activates the non-target evidence penalty.

**Remark** **2** (Post-Annealing Descent to Stationarity)**.** *Assuming the fixed post-annealing objective is bounded below and has an L-Lipschitz gradient, deterministic gradient descent with a sufficiently small step size will eventually converge to first-order stationarity (${∥\nabla F\left(\Theta_{n}\right)∥}_{2}\to 0$). However, this establishes geometric behavior under ideal conditions rather than guaranteeing convergence for the exact optimizer. Because our experiments utilize mini-batch Adam on a nonconvex, piecewise-smooth network, the empirical optimization trajectory can deviate significantly from deterministic gradient descent. We acknowledge this discrepancy and treat the observed variation across random seeds as critical evidence of the model’s empirical stability.*

### 3.5. Dataset Configuration and Stratified Sampling

The theoretical formulation is evaluated using the public four-class peripheral-blood-smear corpus of Ghaderzadeh et al. [51]. The source contains 3562 images (Benign: 502, Early B-ALL: 985, Pre B-ALL: 963, Pro B-ALL: 1112) from 89 subjects. A fixed 80/20 image-level split is constructed with random state 42 and stratification by image label. This split is held constant across all optimization seeds so that between-seed variation reflects stochastic training rather than different validation partitions.

**Remark** **3** (Unit-of-Analysis Limitation)**.** *The available primary-corpus files do not provide patient identifiers or a patient-to-image crosswalk. Consequently, the fixed 80/20 split groups images rather than patients, and images from the same subject may occur in both training and validation partitions. We cannot verify that the split is patient-disjoint, so potential within-patient leakage remains unresolved. Because all five optimization seeds use this same fixed image-level partition, the seed repetitions do not address this risk. Internal validation and checkpoint-selection results should therefore be interpreted as image-level estimates, not evidence of patient-independent performance. Resolving the issue requires patient-to-image identifiers from the data custodian, followed by patient-grouped splitting and retraining; without that information, this limitation cannot be eliminated retrospectively.*

The external evaluation uses the distinct four-class corpus described by Hosseini et al. [52], containing 3242 images (Benign: 512, Early B-ALL: 810, Pre B-ALL: 915, Pro B-ALL: 1005). External labels are never used for optimization or checkpoint selection. The folder names are mapped explicitly to the common indices Benign, Early, Pre, and Pro, and the code verifies that the mapping is bijective. Both collections originate from Tehran; the external collection therefore provides repository-level rather than geographically independent evaluation [50]. Overlap in patient characteristics, demographics, or clinical acquisition protocols between the two datasets cannot be definitively ruled out from the available metadata, limiting the extent to which these results establish generalizability across populations.

### 3.6. Image Transformation and Data Loading

All images are resized to 224×224 pixels. Training images undergo random horizontal reflection and a random rotation of at most $15^{\circ }$. Validation and external images are resized without stochastic augmentation. After conversion to tensors, all images are normalized channelwise using the ImageNet statistics

(29) μ=(0.485,0.456,0.406), σ=(0.229,0.224,0.225).

For a pixel channel value v∈[0,1], normalization applies $v^{\prime }=\left(v-\mu_{c}\right)/\sigma_{c}$. The use of ImageNet normalization is consistent with the pretrained backbone. Data loaders use batch size 32 and four worker processes. Five optimization seeds are used: 42, 123, 456, 789, and 1024. Python (version 3.12.13), NumPy (version 2.0.2), PyTorch (version 2.10.0+cu128), and all available CUDA devices are reset before every model fit; deterministic cuDNN execution is enabled and benchmarking is disabled.

### 3.7. Comparative Model Architectures

All four methods use the same ImageNet-pretrained ResNet-18 [53] architecture and a four-logit output layer, but each model is trained separately. The primary model uses the annealed EMSE–KL objective. The EDL ablation removes the KL term while retaining the evidential mapping and EMSE. The softmax baseline is trained with categorical cross-entropy and uses predictive entropy for error ranking. The Monte Carlo dropout model inserts dropout with rate 0.5 before the final linear layer, is also trained with cross-entropy, and averages 20 stochastic probability vectors at inference. Its uncertainty score is mutual information, computed as predictive entropy minus mean per-pass entropy [15].

At inference, the MC-dropout model is kept in evaluation mode, and only the dropout layer before the final linear layer is selectively enabled to sample dropout masks. Batch-normalization layers therefore remain in evaluation mode and use their running statistics; their statistics are not updated across stochastic passes. For each input, we perform 20 forward passes with independently sampled dropout masks, obtain a softmax probability vector from each pass, and use their arithmetic mean as the predictive probability vector. We compute the uncertainty score as the mutual information, $\mathrm{MI}=H\left(\bar{p}\right)-\frac{1}{20}\sum_{t=1}^{20}H\left(p^{\left(t\right)}\right)$, where $\bar{p}$ is the mean probability vector and *H* denotes categorical entropy.

The backbone architecture and ImageNet-pretraining scheme are held constant, while model parameters are trained independently for each method and seed. This design focuses the comparison on the objective and uncertainty score under a common architecture; it does not test architecture sensitivity or support architecture-independent empirical conclusions.

For an input *x*, the evidential network executes the sequence

(30) $$x\overset{\;f_{\Theta }\;}{\to }z\overset{\;\mathrm{softplus}\;}{\to }e\overset{\;+1\;}{\to }\alpha \overset{\;/S\;}{\to }p,\;u=\frac{K}{S}.$$

Softplus is strictly positive and continuously differentiable, avoiding the zero-gradient region that a ReLU evidence map would create for negative logits. At prediction time, the class label is $\arg\max_{k}p_{k}$, while *u* is retained as a separate scalar reliability indicator.

### 3.8. Optimization, Ablation, and Checkpoint Selection

Every model is trained for 20 epochs with Adam at a learning rate of $10^{-4}$ [37]. The evidential loss uses K=4 and an annealing horizon of 10 epochs. Within each seed, each model is reinitialized after the same seed reset and trained on the same fixed image-level split. The checkpoint with the highest internal validation accuracy is retained. As an auxiliary checkpoint-selection sensitivity analysis, seed 42 was used to compare the checkpoint maximizing validation accuracy with the checkpoint minimizing validation Brier score for each method. Both checkpoints were retained from the same training run, and each was evaluated on the external clean set and on severity-2 blur, brightness, stain, and noise conditions. External outcomes were not used for checkpoint selection. This single-seed analysis is descriptive and does not estimate variability across optimization seeds.

Algorithm 1 summarizes the executable training and evaluation procedure.
**Algorithm 1** Five-seed comparative training and corruption evaluation.**Require:** Seeds S={42,123,456,789,1024}, model definitions, and 13 external conditions
  1:**for all** s∈S **do**  2:      **for all** model m∈{EDL, softmax, MC dropout, EDL without KL} **do**  3:         Reset all random generators to *s* and initialize an ImageNet-pretrained ResNet-18  4:         **for** t=0 **to** 19 **do**  5:               Train with Adam; for EDL use $\lambda_{t}=\min\left(1,t/10\right)$  6:               Retain the checkpoint when validation accuracy improves  7:         **end for**  8:      **end for**  9:      **for all** clean or corrupted external condition *d* **do**10:         Apply the same realized batch to every model and store probabilities, uncertainty scores, and elapsed inference time11:         Compute discrimination, calibration, and selective-prediction statistics12:      **end for**13:**end for**14:**return** the complete seed–model–condition result table


### 3.9. External Corruption Stress Test

The clean external transform uses deterministic resizing, tensor conversion, and ImageNet normalization. Four corruption families are applied at severity levels s∈{1,2,3}. These controlled perturbations are motivated by possible variations in blood-smear images: blur, brightness, stain, and additive Gaussian noise. They are stress tests, not validated simulations of specific microscope hardware or acquisition protocols. Gaussian blur uses a standard deviation sampled uniformly from [0.5,0.5+0.5s]. Brightness uses ColorJitter with brightness=0.2s; stain shift uses ColorJitter with hue=0.1s and saturation=0.1s; and Gaussian noise adds a tensor sampled from $N(0,{\left(0.05s\right)}^{2})$, with pixel values clipped to [0,1]. The transformations are implemented using torchvision.transforms. Together with the clean condition, this produces 13 test conditions.

Within a data-loader pass, the same transformed image batch is supplied to every model. A deterministic seed is assigned to each image–corruption-family–severity combination. This includes the sampled blur parameter and the stochastic brightness, stain, and noise transforms, ensuring that all models are evaluated on the same realized corrupted image for each condition. Corrupted images are regenerated at evaluation time rather than stored as separate image files.

### 3.10. Uncertainty and Calibration Metrics

Evaluation combines class decisions, ranking performance, proper probabilistic scores, and uncertainty diagnostics. The primary discrimination measures are accuracy, balanced accuracy, macro-$F_{1}$, macro one-versus-rest AUROC, mean sensitivity, and mean specificity. For class *k*, precision, recall, and $F_{1}$ are

(31) $${\mathrm{Precision}}_{k}=\frac{{\mathrm{TP}}_{k}}{{\mathrm{TP}}_{k}+{\mathrm{FP}}_{k}},\;{\mathrm{Recall}}_{k}=\frac{{\mathrm{TP}}_{k}}{{\mathrm{TP}}_{k}+{\mathrm{FN}}_{k}},\;F_{1,k}=\frac{2{\mathrm{Precision}}_{k}{\mathrm{Recall}}_{k}}{{\mathrm{Precision}}_{k}+{\mathrm{Recall}}_{k}}.$$

Macro averages weight classes equally, whereas weighted averages use empirical class support. One-versus-rest receiver operating characteristic curves evaluate the ranking induced by each $p_{k}$, and the area under each curve summarizes threshold-independent discrimination.

Reliability is evaluated using the multiclass Brier Score and ECE. For *N* samples, the Brier Score is

(32) $$\mathrm{BS}=\frac{1}{N}\sum_{i=1}^{N}\sum_{k=1}^{K}{\left(p_{ik}-y_{ik}\right)}^{2}.$$

The Brier Score is a strictly proper scoring rule for categorical probabilities [19,20]. Under the convention in Equation (32), its range is [0,2] for one-hot multiclass outcomes. It jointly reflects discrimination and calibration and therefore should not be interpreted as a pure calibration statistic.

For ECE, predictions are partitioned into *M* confidence bins $B_{m}$. The metric is

(33) $$\mathrm{ECE}=\sum_{m=1}^{M}\frac{|B_{m}|}{N}\left|\mathrm{acc}\left(B_{m}\right)-\mathrm{conf}\left(B_{m}\right)\right|.$$

The implementation uses M=10 equal-width bins and top-1 confidence $c_{i}=\max_{k}p_{ik}$. Empty bins contribute zero. For expected calibration error (ECE), we emphasize that the metric is highly sensitive to the chosen binning strategy and can be unstable in finite samples. We report ECE for descriptive comparison alongside the strictly proper Brier Score, which does not rely on arbitrary binning parameters.

For selective prediction, samples are sorted by decreasing top-1 confidence, as formalized in Section 3.3. At coverage *q*, selective risk is the cumulative error rate among the retained predictions, and the area under this curve is denoted AURC. Error-detection AUROC treats an incorrect prediction as the positive class and ranks samples by the model-specific uncertainty score.

For each model pair, condition, and selected metric (accuracy, Brier score, ECE, AURC, and error-detection AUROC), we conduct a paired Wilcoxon signed-rank test across the five matched optimization seeds. Exact two-sided *p*-values are calculated from all sign assignments to the observed nonzero rank scores, using average ranks for tied absolute differences. Holm’s step-down procedure is applied once across the complete family of 390 tests (6 model pairs × 13 conditions × 5 metrics). Two-sided *t*-based 95% confidence intervals for each model–condition mean are computed across seeds. These intervals quantify optimization-seed variation conditional on the fixed image split and external images; with only five seeds, they are descriptive and are not patient-level or population-sampling intervals. As ordinary *t* intervals, they are not constrained to each metric’s natural range. The smallest attainable two-sided exact Wilcoxon *p*-value with five nonzero paired differences is 0.0625; accordingly, this design cannot establish significance at the 0.05 level, even before multiplicity correction. These tests are exploratory, and nonsignificance is not evidence of equivalence.

### 3.11. Implementation Fidelity and Reproducibility Checklist

Table 2 records the implemented experimental settings. Values not represented in the code, such as weight decay, a learning-rate scheduler, early stopping patience, or test-time augmentation, are not assumed.

## 4. Results

**Figure 2:**
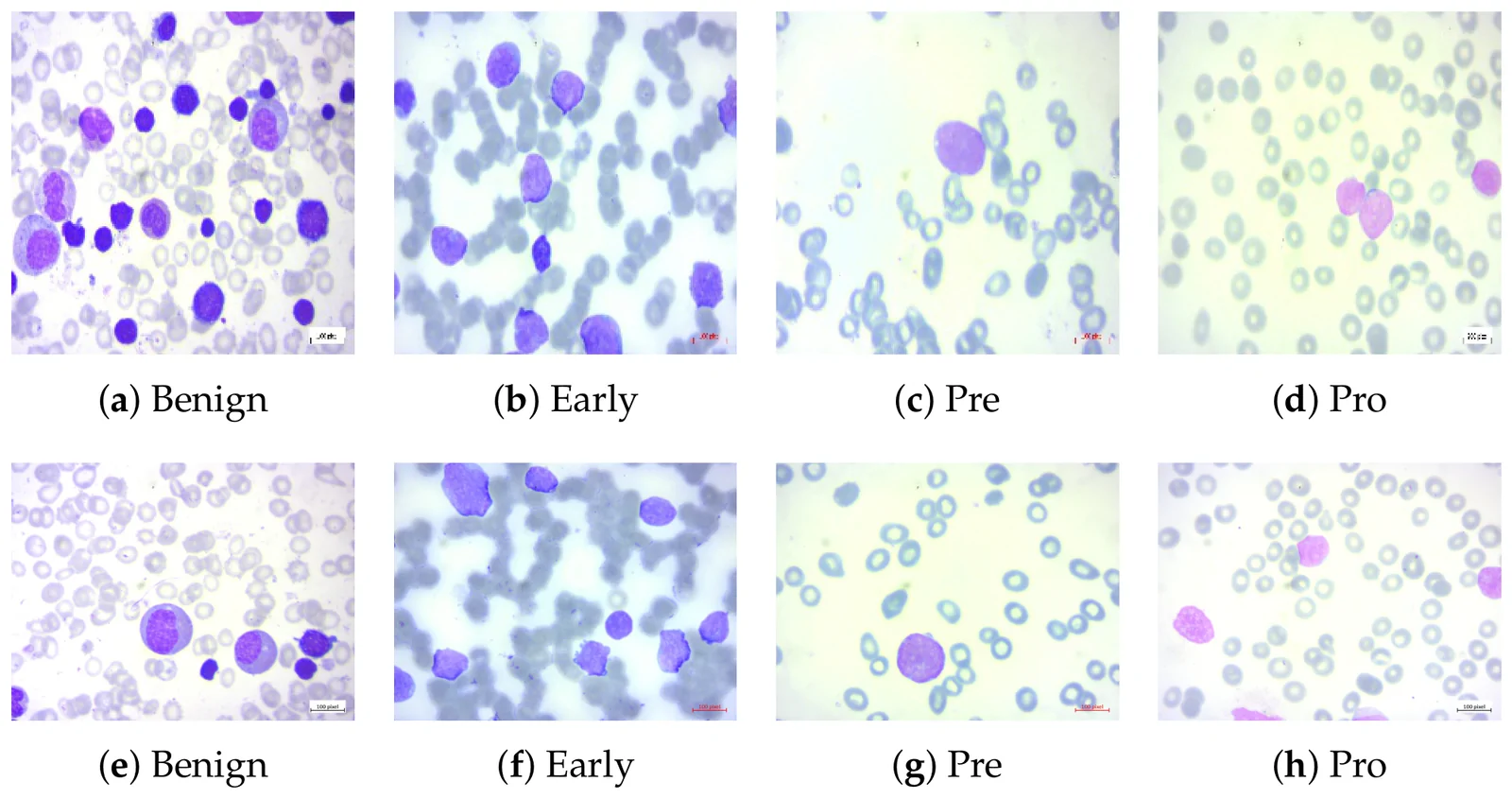
Representative blood-smear images illustrating the four classes from the two studied datasets. Top row: (**a**) Benign, (**b**) Early, (**c**) Pre, and (**d**) Pro from Ghaderzadeh et al. [51]. Bottom row: (**e**) Benign, (**f**) Early, (**g**) Pre, and (**h**) Pro from Hosseini et al. [52].

The four-class experiment assesses how changes in the external input environment affect the prediction-and-selection pipeline; representative images from the four classes are shown in Figure 2. We report predictive discrimination, calibration, confidence-ranked selective risk, uncertainty-ranked error detection, corruption sensitivity, and computational cost. The mathematical results describe constraints on the representation; the experiments evaluate behavior under the specified shifts without extending those constraints into a guarantee of decision reliability.

### 4.1. Analytical Consequences for the Four-Class Setting

Before considering the numerical experiment, the general results of Section 3 can be specialized to K=4. Table 3 summarizes the domains implied by the softplus and unit-prior construction.

The general EMSE bound becomes

(34) $$0\leq L_{\mathrm{EMSE}}\leq 2+\frac{3}{4\cdot 5}=2.15.$$

At the no-evidence limit $\alpha_{k}=1$, the model has $p_{k}=1/4$ and u=1. For any one-hot target, the squared-error term is 3/4 and the summed variance term is 3/20, giving

(35) $$L_{\mathrm{EMSE}}\left(1,y\right)=0.75+0.15=0.90.$$

This value provides a useful reference: a sample whose EMSE remains near 0.90 behaves like an uninformative four-class opinion, whereas a correctly oriented high-evidence sample can approach zero loss.

For a correct evidence ray, let r=y and α(c)=1+cy. Direct substitution yields

(36) $$L_{\mathrm{EMSE}}\left(c\right)=\frac{12}{{\left(c+4\right)}^{2}}+\frac{6(c+2)}{{\left(c+4\right)}^{2}\left(c+5\right)},\;u\left(c\right)=\frac{4}{c+4}.$$

Both expressions decrease to zero. The first term in Equation (36) is the squared error of the mean and the second is the Dirichlet variance. Thus, even after the class decision is correct, increasing target evidence continues to decrease the objective by sharpening the distribution. Conversely, if the evidence ray points entirely toward a wrong class, the variance still vanishes but the squared-error term approaches its maximum value of 2. Masking the KL term contracts the latter type of competing evidence, but Theorem 2 shows that it leaves the correct target ray unregularized.

For K=4, Equations (13) and (14) become

(37) $$c\leq 1-\frac{3}{4}u,\;{∥y-p∥}_{2}^{2}\geq \frac{3}{4}u^{2}.$$

Hence an uncertainty value of u=0.2, for example, restricts confidence to at most 0.85 and contributes a Brier floor of 0.03 even in the best-oriented opinion. This is an algebraic constraint on projected probabilities, not evidence of underconfidence or a causal explanation of the observed calibration scores.

For K=4, the local KL Hessian in Equation (23) has tangential eigenvalue $\psi_{1}\left(1\right)=\pi^{2}/6\approx 1.6449$ and full radial eigenvalue $\psi_{1}\left(1\right)-4\psi_{1}\left(4\right)\approx 0.5096$. Both are positive. The regularizer is therefore locally restoring in every concentration direction around the unit Dirichlet, although contraction is weaker in the common radial direction than in directions that redistribute concentration among classes.

The exact annealing coefficients implied by the implementation are shown in Table 4. This table is included because the displayed epoch number is one greater than the zero-based epoch index passed to the loss function.

### 4.2. Clean External Performance and Computational Cost

Table 5 reports means and standard deviations across five optimization seeds. All four methods achieve near-ceiling clean accuracy (0.9984–0.9992), so accuracy alone does not discriminate between them on this external clean condition. Softmax has the lowest mean Brier score (0.0016) and ECE (0.0030). Softmax has the highest error-detection AUROC (0.9983) and the lowest AURC (0.0000016). These rankings differ by reliability target. Across all 390 paired tests, no raw *p*-value is below 0.05 and no Holm-adjusted comparison is significant. The minimum raw exact two-sided *p*-value is 0.0625.

A separate synchronized checkpoint benchmark measured inference on CUDA using batches of 32 images, 10 warm-up repetitions, and 50 timed repetitions per seed and model. Table 6 reports the mean and standard deviation across five seed-specific checkpoints. The timed region includes model forward passes and predictive probability/uncertainty scoring, but excludes image loading, preprocessing, and host-to-device transfer. The three single-pass methods require approximately 0.93–0.94 ms per image, whereas 20-pass MC dropout requires 18.875 ms per image, approximately 20.2 times the mean single-pass latency. These are model-stage timings rather than end-to-end pipeline latency.

### 4.3. Checkpoint-Selection Sensitivity (Seed 42)

For all four methods, both selected checkpoints attained validation accuracy 1.000; minimizing validation Brier selected a later epoch and a lower validation Brier score in each case (Table 7). The external differences were mixed and depended on method, metric, and corruption. Differences below are Brier-selected minus accuracy-selected, so the sign should be interpreted according to each metric’s direction. For softmax under noise severity 2, Brier-based selection changed accuracy by +0.0839, Brier score by −0.2287, ECE by −0.1066, AURC by −0.1338, and error-detection AUROC by +0.1411. In contrast, for EDL without KL under the same condition, the corresponding differences were −0.0234, +0.0232, +0.0084, +0.1727, and −0.0638. For EDL with masked KL under stain severity 2, accuracy increased by 0.0170, while Brier score, ECE, and AURC increased by 0.0175, 0.0372, and 0.0428, respectively, and error-detection AUROC decreased by 0.0779. Thus, the alternative criterion changed some external outcomes substantially but did not produce a uniformly preferable checkpoint. These results are descriptive for one seed and five external conditions, not evidence of cross-seed stability or a basis for inferential claims. For both EDL methods, clean error-detection AUROC differences were unavailable because at least one selected checkpoint made no clean-set errors.

### 4.4. Accuracy Degradation Under External Corruptions

The severity-averaged results in Table 8 show that accuracy rankings depend on corruption family. The no-KL ablation has the highest mean accuracy under blur (0.989) and noise (0.526), softmax leads under brightness (0.894), and MC dropout leads under stain (0.723). MC dropout drops to 0.471 mean accuracy under noise.

**Figure 3:**
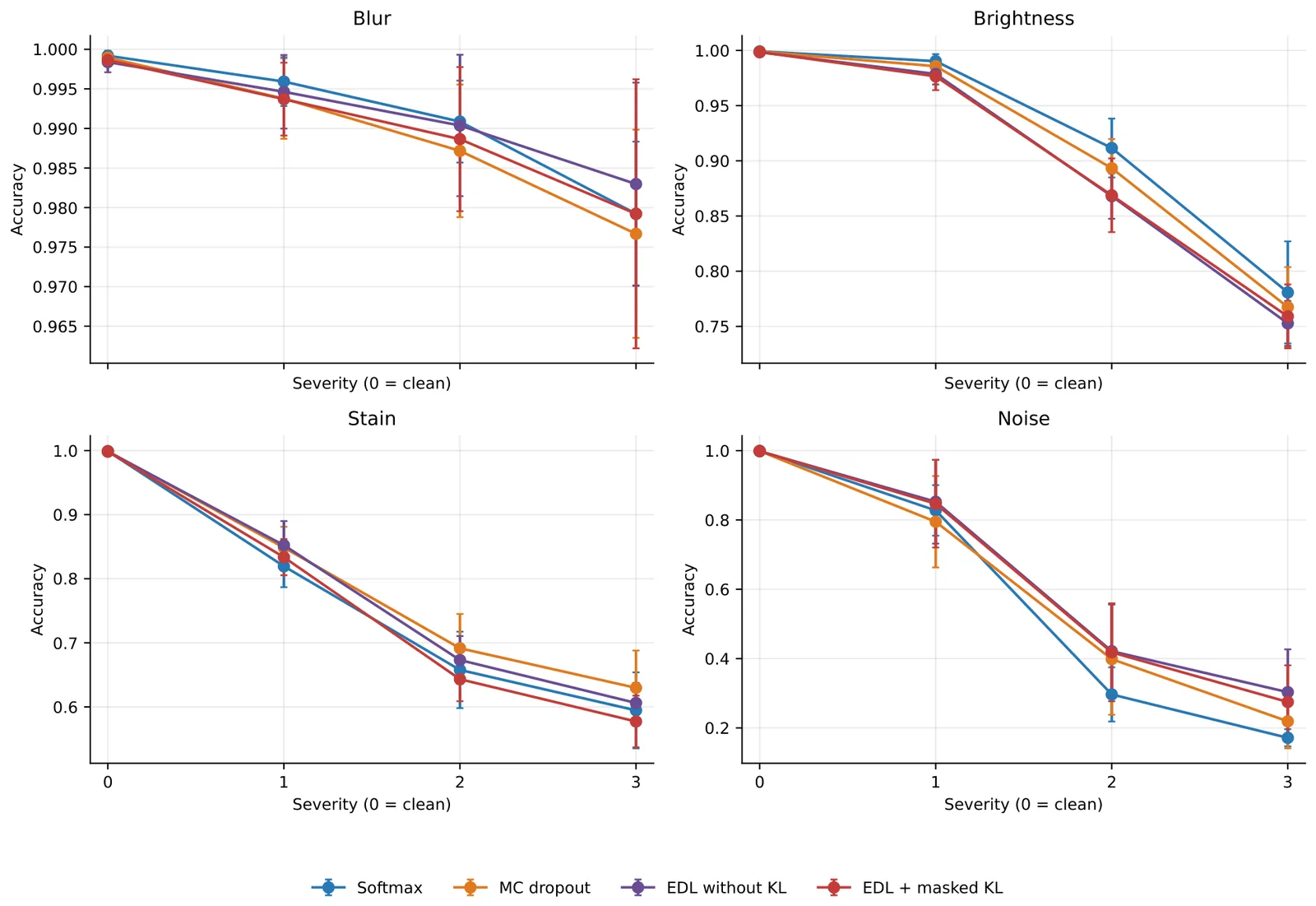
External accuracy versus corruption severity for blur, brightness, stain, and Gaussian noise. Points and error bars denote the mean and standard deviation across five optimization seeds; severity 0 is the clean external cohort.

Figure 3 shows that blur has little effect on any method, whereas brightness and Gaussian noise cause marked severity-dependent losses. Equal weighting of the four corruption families and severities 1–3 gives mean accuracies of 0.751 for softmax, 0.766 for MC dropout, 0.773 for EDL without KL, and 0.763 for regularized EDL. This descriptive aggregate favors the no-KL ablation but does not encode real-world corruption frequencies or operational risk.

### 4.5. Uncertainty Quantification and Calibration Analysis

**Figure 4:**
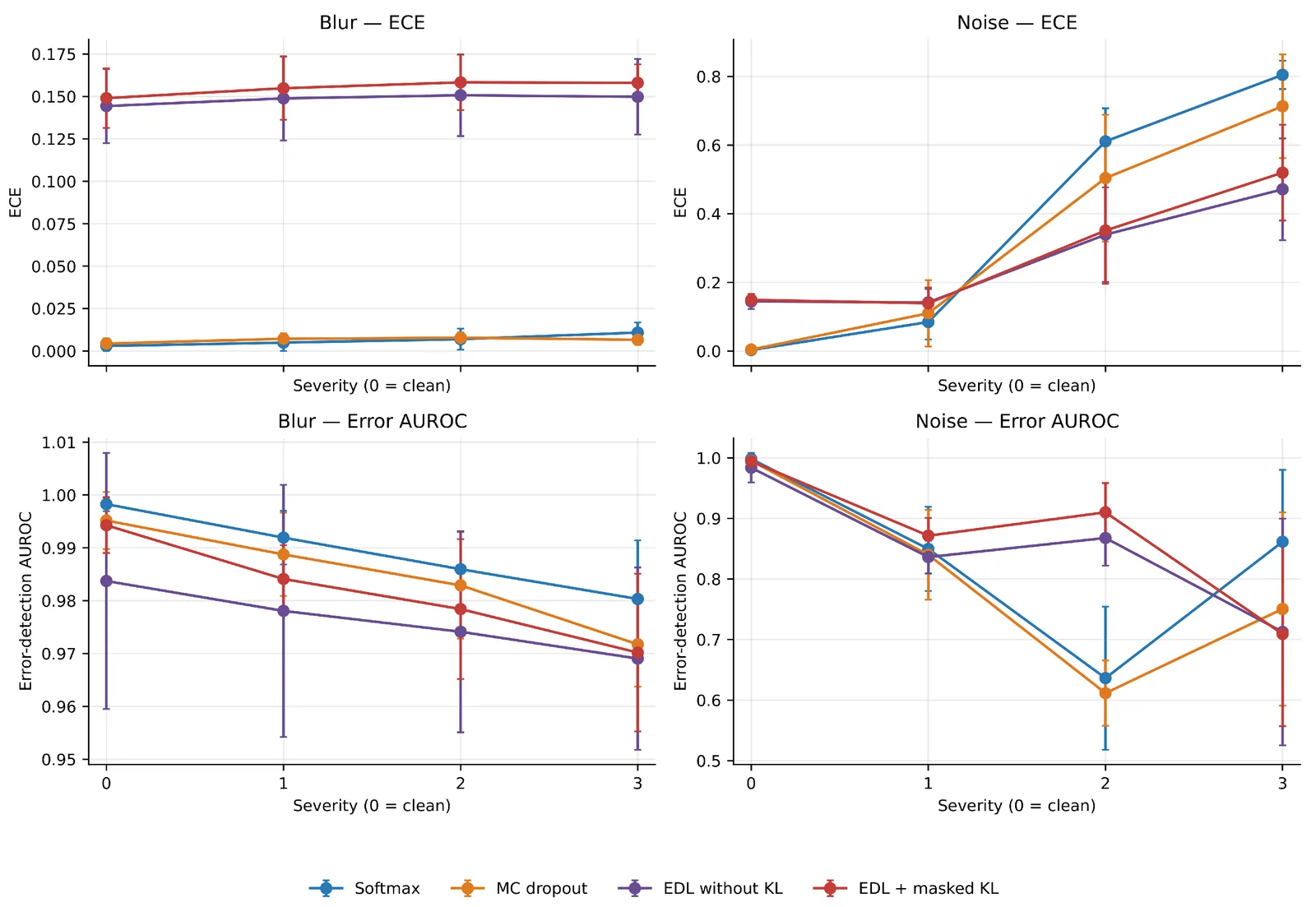
Expected calibration error (**top**) and error-detection AUROC (**bottom**) under blur and Gaussian-noise corruption across severity levels. Points and error bars denote mean and standard deviation across five optimization seeds; severity 0 is the clean external cohort.

ECE and error-detection AUROC do not yield a common model ranking. Under blur, MC dropout has the lowest ECE (0.007), while softmax has the highest error-detection AUROC (0.986). Under brightness, MC dropout again has the lowest ECE (0.061), while softmax has the highest error-detection AUROC (0.945). Under stain, EDL without KL has the lowest ECE (0.112), while MC dropout has the highest error-detection AUROC (0.770). Under noise, EDL without KL has the lowest ECE (0.317), whereas regularized EDL has the highest error-detection AUROC (0.830). Figure 4 shows that ECE and error-detection AUROC vary differently across blur and noise severities. Probability calibration and uncertainty-based error ranking must therefore be evaluated separately.

**Figure 5:**
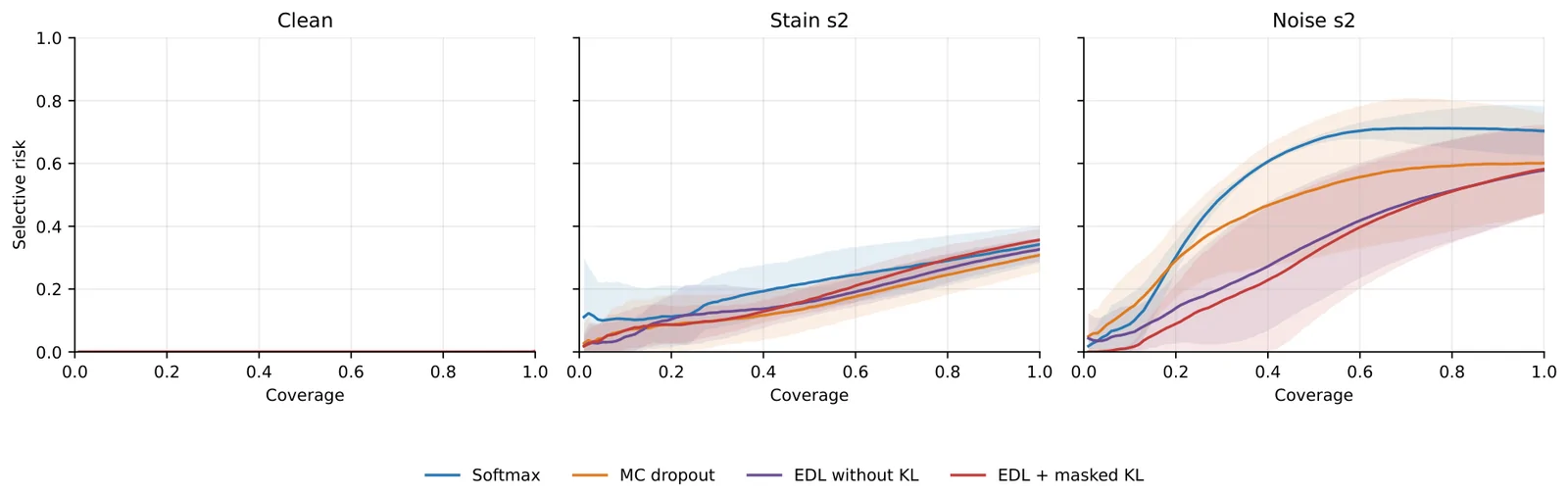
Confidence-ranked selective risk–coverage curves for clean, stain-severity-2, and noise-severity-2 external conditions. Curves are computed separately for each seed from per-image maximum-probability confidence; lines show the mean and shaded bands show ±1 standard deviation across five optimization seeds.

The clean AURC values are near zero for every model because clean accuracy is near one; softmax has the smallest mean AURC (0.0000016), while EDL without KL has the largest (0.0000075). Figure 5 presents confidence-ranked risk–coverage curves for clean, stain-severity-2, and noise-severity-2 conditions. Curves are computed per seed from the saved image-level predictions and summarized across the five optimization seeds. These retrospective summaries do not validate a clinical referral threshold or downstream workflow.

**Figure 6:**
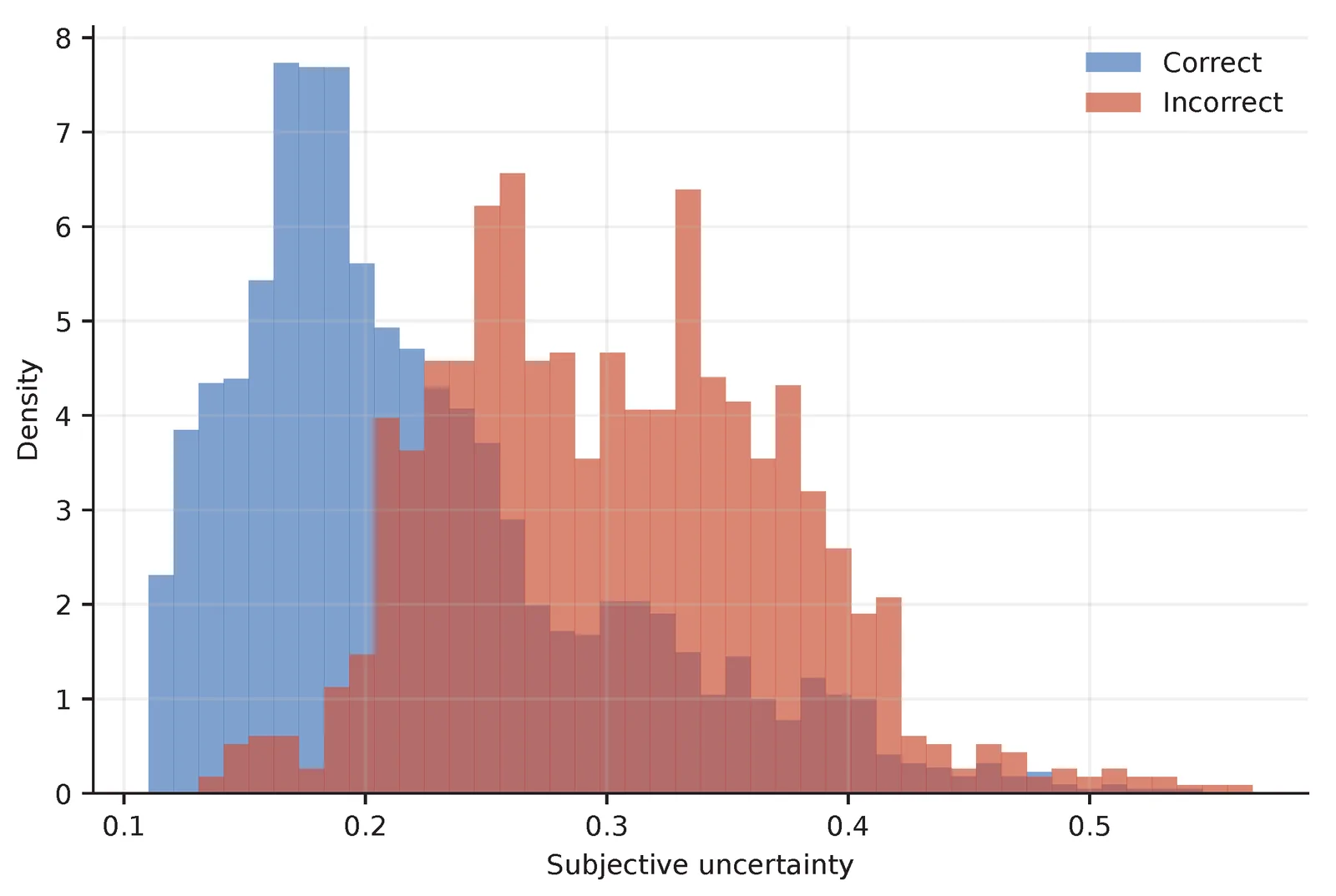
Subjective-uncertainty density among correct and incorrect EDL-with-masked-KL predictions for seed 42 under stain-severity-2 corruption. This single-seed plot is illustrative and is not an across-seed inferential comparison.

Clean error-detection AUROC ranges from 0.9837 for EDL without KL to 0.9983 for softmax, with regularized EDL at 0.9943 and MC dropout at 0.9952. Figure 6 adds an illustrative distribution of the EDL-with-masked-KL uncertainty score for correct and incorrect predictions under stain severity 2. This plot uses seed 42 only and is descriptive; error-detection AUROC remains the across-seed quantitative summary.

**Figure 7:**
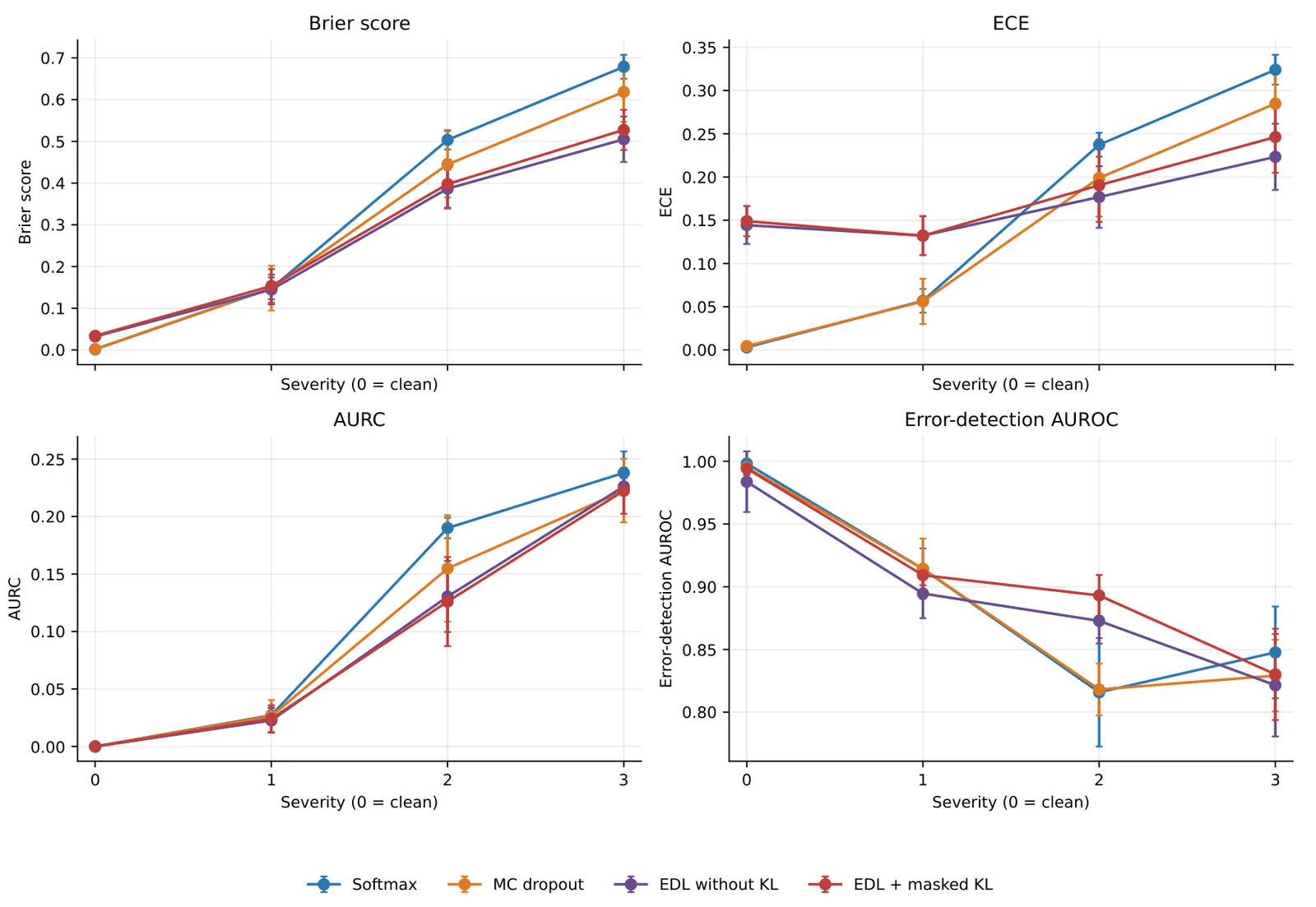
Direct ablation of the masked KL penalty, averaged equally across corruption families at each severity level. Curves compare regularized EDL with EDL without KL; points and error bars show the mean and standard deviation across five seeds. Lower values are preferable for Brier score, ECE, and AURC; higher values are preferable for error-detection AUROC. Severity 0 denotes the clean cohort.

Across all corruption conditions with equal weighting, regularized EDL minus EDL without KL gives mean differences of −0.0097 in accuracy, +0.0140 in Brier score, +0.0122 in ECE, −0.0022 in AURC, and +0.0145 in error-detection AUROC. Thus, the no-KL model has better averaged accuracy, Brier score, and ECE, whereas regularized EDL has slightly lower averaged AURC and higher error-detection AUROC. These are descriptive differences, not statistically established effects. Figure 7 summarizes the condition-dependent pattern across severity.

For the noise-severity-2 Brier comparison, the paired seed-wise difference (regularized EDL minus EDL without KL) is −0.0024±0.0496 (mean ± SD). The exact Wilcoxon test gives raw p=0.8125 and Holm-adjusted p=1.000. This small-sample result does not establish a consistent Brier-score benefit or equivalence between the methods.

The empirical results provide mixed rather than uniformly favorable evidence for masked KL. This does not change the mathematical result: the penalty acts on non-target evidence but leaves the target-evidence direction unregularized. Its effect on predictive reliability must therefore be established empirically for each condition and metric rather than presumed from the regularizer’s local geometry.

## 5. Discussion

This section connects the representation-level results to the prediction and selection pipeline. We first interpret loss geometry and optimization, then examine the observed reliability trade-offs and their consequences for prediction retention. The distinction between a subjective uncertainty representation, an empirical error-ranking signal, and a validated decision policy is central to this interpretation.

### 5.1. Loss Geometry and Stability

The analysis separates the predictive direction p=α/S from the evidence scale *S*. The EMSE combines a Brier loss at the Dirichlet mean with posterior variance, so it rewards both correct direction and increasing concentration. The confidence–uncertainty envelope makes the resulting trade-off explicit: retained uncertainty imposes an upper bound on confidence and a quadratic floor on the Brier component.

The masked KL term has an exact but limited role. Its unique unit-Dirichlet minimum and positive local curvature contract non-target evidence. However, Theorem 2 shows that masking also makes the penalty identically zero along the pure target-evidence ray. The complete regularized loss is therefore noncoercive and has no finite preferred target concentration. This is stronger than observing evidence inflation in the unregularized EMSE: the standard masked KL construction does not remove that radial degeneracy.

Consequently, a small evidential uncertainty value should not be interpreted as an unconditional guarantee of correctness or distance from the training distribution. It states that the network produced a large total concentration. Whether that concentration is trustworthy remains an empirical question under distribution shift [7,22].

### 5.2. Annealing and the Scope of Convergence

Annealing is used because the EMSE and KL terms have different early-training roles. At initialization, predictions are poorly oriented and many samples contain evidence for several classes. Applying the full KL penalty immediately can contract the evidence vector before the feature extractor has learned discriminative directions. The linear schedule gives the EMSE a finite period in which to organize class structure, then progressively increases the penalty on misleading evidence. This interpretation is consistent with empirical work emphasizing annealed Dirichlet regularization [12,18]; the present experiments do not establish that annealing is necessary or that the chosen schedule is optimal.

The article distinguishes concentration-space geometry, parameter-space optimization, and empirical stability. The KL minimizer and the target-ray noncoercivity result are exact concentration-space statements. Post-annealing deterministic gradient descent reaches first-order stationarity only under the stated smoothness and step-size assumptions. The experiments instead use mini-batch Adam on a nonconvex, piecewise-smooth network [37,38]. The observed seed variation, particularly under noise, is therefore important evidence rather than a nuisance to suppress.

### 5.3. Reliability Trade-Offs Under Corruption

The corrected clean results show near-ceiling accuracy for all four methods. Softmax has the lowest Brier score, ECE, and AURC, and also the highest error-detection AUROC. Neither ECE nor Brier score alone establishes underconfidence, and the algebraic Brier floor should not be treated as a causal explanation of the empirical calibration differences.

The rankings change across corruption families. Across the 390 paired tests, no raw comparison is significant and every Holm-adjusted *p*-value is 1.0. The noise-severity-2 Brier difference is −0.0024±0.0496 (regularized minus no-KL), with raw p=0.8125 and adjusted p=1.0. This reflects the small number of seeds and multiplicity correction; it is not evidence that the methods are equivalent.

The ablation also tempers the mechanistic claim. Masked KL lowers selected Brier and ECE values but does not consistently improve accuracy, AURC, or error-detection AUROC across severity levels. This agrees with its mathematical scope: removing non-target evidence can alter probability quality and uncertainty ordering without establishing a finite target-evidence scale or shift-independent robustness.

### 5.4. Implications for Medical-Image Classification

The mathematical constraints connect the predictive representation to the retention interface. By Equation (13), accepting a prediction at confidence c≥τ necessarily implies u≤K(1−τ)/(K−1). The converse does not hold: low subjective uncertainty need not imply high top-1 confidence, and neither condition guarantees correctness. Thus, an uncertainty threshold cannot simply be substituted for the confidence ordering used to calculate AURC. The noncoercivity result further shows why increasing evidence strength is not, by itself, evidence that a retained prediction is trustworthy.

At the pipeline level, a change in input conditions can alter both predictive probabilities and the ordering used to retain predictions. Calibration, error-detection AUROC, and selective risk therefore answer complementary questions; improvement in one is not proof of improvement in the others. The clean results illustrate this distinction: softmax has the lowest ECE and AURC and the highest error-detection AUROC. The computational comparison describes inference cost within the tested implementation, not the total cost of a service with human review.

The present formulation predicts one Dirichlet distribution per image. It does not implement a credal-set decision rule, interval-valued predictions, or worst-case optimization over an ambiguity set. Its relevance to epistemic decision support lies in examining the limitations of a commonly used higher-order representation. In particular, the experiments assess whether the subjective uncertainty mass helps rank errors; they do not identify ground-truth epistemic uncertainty or establish an empirical separation from aleatoric uncertainty.

A deployable retention rule would require a threshold chosen independently of the external test outcomes, evaluation of retained risk and coverage under anticipated shifts, and explicit treatment of the consequences of abstention. Human referral capacity, asymmetric error costs, and monitoring of changing input conditions would enter that broader system design. These are requirements for future validation rather than capabilities demonstrated by the present experiment.

### 5.5. Limitations and Future Work

The distributed primary data do not provide patient identifiers or an image-to-patient crosswalk, so we cannot verify that the fixed partition is patient-disjoint. Images from the same patient may occur in both training and validation sets, creating a risk of optimistic validation estimates and affecting checkpoint selection. Repeating optimization across five seeds does not resolve this issue because every seed uses the same fixed image-level split. The reported validation and model-selection results therefore do not establish patient-independent performance. A definitive correction requires patient-to-image identifiers from the data custodian and a rerun using patient-grouped partitions; if these identifiers remain unavailable, this risk must be acknowledged as unresolved.

Brightness, stain, and noise corruptions are generated from fixed per-image seeds rather than archived corrupted tensors. Every model receives the same deterministic corruption for each image and condition. The separate synchronized CUDA-event benchmark reports model-stage latency only; it excludes image loading, preprocessing, and host-to-device transfer, so it should not be interpreted as end-to-end pipeline latency. The statistical analysis supplies across-seed means and standard deviations, two-sided *t*-based 95% intervals, and 390 paired Wilcoxon tests with Holm correction. These results remain conditional on one image-level split. The smallest raw exact two-sided *p*-value was 0.0625, so no comparison could reach 0.05 even before multiplicity correction.

The experiments compare four methods using one backbone architecture, one fixed split, and one external image collection. The primary five-seed analysis selected checkpoints by validation accuracy, and we additionally compared validation-accuracy and validation-Brier selection for seed 42 on the clean set and four severity-2 corruptions. This exploratory comparison showed condition- and metric-dependent changes, but one seed and five external conditions cannot establish the stability of reliability metrics to checkpoint choice across seeds or corruption severities. The main comparative results should therefore not be interpreted as robust to selection choices. A follow-up should prespecify alternative criteria using validation data only and evaluate the resulting checkpoints on the untouched external set without further tuning. The risk–coverage curves cover three conditions and do not establish operating thresholds. Temperature scaling, deep ensembles, Prior Networks, learned base rates, patient-level resampling, and controlled out-of-distribution datasets are also not evaluated [5,16,28]. No downstream clinical outcomes, referral workload, or closed-loop adaptation are evaluated.

The external collection is used only for testing, so the study still has one development corpus; it does not provide a second training-dataset replication or geographically independent validation. The five optimization seeds quantify variability conditional on the same image-level split and architecture, not variability across patient partitions, cohorts, or model families. Accordingly, the empirical findings are limited to this controlled blood-smear case study and should not be read as broad experimental validation of EDL across image-classification tasks.

Assessing broader empirical generality will require replication across architectures and development cohorts, together with repeated or cross-validated partitions. Patient-disjoint evaluation would additionally require image-to-patient identifiers, which are unavailable in the distributed data.

Future mathematical work should study regularizers that act on target strength or total concentration without collapsing useful discrimination, thereby addressing the identified noncoercive direction. Any proposed finite-scale objective would require its own existence and optimization analysis, followed by evaluation of whether it improves both uncertainty interpretation and retained prediction risk. A finite evidence scale alone would not establish robust decision-making.

## 6. Conclusions

This article combines mathematical analysis of evidential uncertainty with an external corruption study in blood-smear image classification. Two mathematical results clarify the limits of the predictive representation. Subjective uncertainty, confidence, and Brier loss are algebraically coupled: retaining uncertainty limits confidence and imposes a nonzero quadratic-loss floor. In addition, masked KL contracts non-target evidence but leaves a target-evidence direction unpenalized, so the complete concentration-space objective is noncoercive and approaches an unattained zero infimum along that ray.

The five-seed stress test shows condition-dependent reliability patterns. Clean accuracy is near one for every method, while softmax has the best mean clean Brier score, ECE, AURC, and error-detection AUROC. Across the 12 corruption conditions, EDL without KL has the highest equal-weighted mean accuracy and lower overall mean Brier score and ECE; regularized EDL has slightly lower mean AURC and higher mean error-detection AUROC. None of the 390 paired comparisons is significant after Holm correction. With five pairs, the smallest attainable two-sided exact Wilcoxon *p*-value is 0.0625, so these tests cannot establish a difference at the conventional 0.05 level, and nonsignificance does not establish equivalence. Confidence-ranked selective risk and uncertainty-based error detection provide complementary retrospective summaries, neither of which establishes a validated deployment threshold. These results do not show a general reliability advantage for masked KL. Patient-aware and geographically independent evaluation, independently selected operating thresholds, and assessment of downstream abstention consequences remain necessary before clinical use can be considered.

## Figures and Tables

**Table 2:** Code-faithful experimental configuration.

Component	Implemented Setting	Interpretive Note
Randomization	Five seeds: 42, 123, 456, 789, and 1024	Fixed split; model optimization is repeated.
Internal split	80/20 image-level stratification, random state 42	Patient-disjoint splitting was impossible because patient identifiers were unavailable.
Input size	224×224 RGB	Matches the standard ResNet input resolution.
Training augmentation	Horizontal flip and rotation in $\left[-15^{\circ },15^{\circ }\right]$	No color or stain augmentation is implemented.
Normalization	ImageNet channel means and standard deviations	Consistent with pretrained weights.
Backbone	ImageNet-pretrained ResNet-18	Final layer replaced by four logits.
Compute hardware	NVIDIA T4 GPU	GPU acceleration was used when CUDA was available.
Optimizer	Adam, learning rate $10^{-4}$	No explicit weight decay or scheduler.
Batching	Batch size 32; four workers	Training shuffled; evaluation not shuffled.
Models	EDL, EDL without KL, softmax, and MC dropout	All use ResNet-18; MC dropout uses 20 passes.
Objectives	EMSE plus masked KL; EMSE only; cross-entropy	KL coefficient ramps from 0 to 1 over zero-indexed epochs 0–10.
Training duration	20 epochs	Best checkpoint selected by validation accuracy.
External conditions	Clean plus four corruptions at three severities	Blur, brightness, stain shift, and Gaussian noise.
Evaluation	Accuracy, balanced accuracy, macro-$F_{1}$/AUROC, sensitivity, specificity, Brier, ECE, AURC, and error AUROC	Across-seed results are summarized by the mean and standard deviation.
Inference	One pass for softmax and EDL; 20 for MC dropout	Dedicated synchronized checkpoint timing is reported separately from full data-pipeline latency.

**Table 3:** Deterministic ranges of the principal evidential quantities for K=4.

Quantity	Range	Interpretation
Evidence $e_{k}$	(0,∞) for finite logits	Softplus produces strictly positive numerical evidence, approaching zero as $z_{k}\to -\infty$.
Concentration $\alpha_{k}$	(1,∞) for finite logits	Unit prior plus learned evidence.
Strength *S*	(4,∞) for finite logits	Total prior and learned evidence.
Mean probability $p_{k}$	(0,1) with $\sum_{k}p_{k}=1$	Projected subjective probability and Dirichlet mean.
Belief mass $b_{k}$	[0,1)	Committed support for class *k*.
Uncertainty *u*	(0,1), with limiting value 1 at zero evidence	Uncommitted subjective mass.

**Table 4:** KL annealing schedule implemented by the training loop.

Displayed Epoch	Code Index *t*	$\lambda_{t}$	Interpretation
1	0	0.0	EMSE only
2	1	0.1	Initial KL activation
6	5	0.5	Equal numerical weighting coefficient
10	9	0.9	Near-full regularization
11	10	1.0	First epoch at full KL strength
20	19	1.0	Full regularization retained

**Table 5:** Clean external performance across five optimization seeds (mean ± standard deviation).

Model	Accuracy	Brier	ECE	AURC	Error AUROC
Softmax	0.9992±0.0003	0.0016±0.0010	0.0030±0.0029	0.000002±0.000002	0.9983±0.0013
MC dropout	0.9990±0.0009	0.0023±0.0021	0.0044±0.0030	0.000006±0.000012	0.9952±0.0054
EDL without KL	0.9984±0.0013	0.0324±0.0089	0.1444±0.0218	0.000007±0.000009	0.9837±0.0242
EDL + masked KL	0.9986±0.0007	0.0340±0.0078	0.1490±0.0176	0.000004±0.000003	0.9943±0.0052

**Table 6:** Synchronized CUDA-event inference latency across five seed-specific checkpoints. Values are milliseconds per image (mean ± standard deviation). The timed region includes forward inference and probability/uncertainty scoring; image loading, transforms, and host-to-device transfer are excluded.

Model	Forward Passes	Latency (ms/Image)
Softmax	1	0.938±0.015
MC dropout	20	18.875±0.449
EDL without KL	1	0.933±0.004
EDL + masked KL	1	0.934±0.007

**Table 7:** Seed-42 checkpoint-selection comparison. Validation Brier is reported for the accuracy-selected and Brier-selected checkpoint, respectively. Both selected checkpoints had validation accuracy 1.000.

Model	Accuracy-Selected Epoch	Brier-Selected Epoch	Validation Brier (Accuracy/Brier)
Softmax	14	16	0.000476/0.000186
MC dropout	5	12	0.001090/0.0000167
EDL without KL	2	18	0.030572/0.009217
EDL + masked KL	2	19	0.030563/0.011923

**Table 8:** Mean performance across five seeds and severities 1–3 within each corruption family.

Corruption	Model	Accuracy	Brier	ECE	Error AUROC
Blur	Softmax	0.989	0.017	0.008	0.986
	MC dropout	0.986	0.021	0.007	0.981
	EDL without KL	0.989	0.049	0.150	0.974
	EDL + masked KL	0.987	0.055	0.157	0.978
Brightness	Softmax	0.894	0.163	0.062	0.945
	MC dropout	0.882	0.175	0.061	0.931
	EDL without KL	0.867	0.200	0.131	0.935
	EDL + masked KL	0.868	0.197	0.136	0.936
Stain	Softmax	0.691	0.558	0.255	0.724
	MC dropout	0.723	0.480	0.210	0.770
	EDL without KL	0.711	0.426	0.112	0.738
	EDL + masked KL	0.685	0.461	0.129	0.766
Noise	Softmax	0.432	1.035	0.500	0.783
	MC dropout	0.471	0.940	0.442	0.734
	EDL without KL	0.526	0.707	0.317	0.806
	EDL + masked KL	0.513	0.724	0.337	0.830

## Data Availability

The datasets analyzed in this study are publicly available through the repositories described in the cited source publications [51,52]. The experiment code is publicly available at https://github.com/umarbhasan/conditional-convergence (accessed on 26 October 2026).

## Supplementary Materials

The following supporting information can be downloaded at https://www.mdpi.com/article/10.3390/s26216957/s1: Supplementary Material S1. proofs of Propositions 1–4.

## Abbreviations

The following abbreviations are used in this manuscript:

AUC	Area Under the Curve
BNN	Bayesian Neural Network
EDL	Evidential Deep Learning
ECE	Expected Calibration Error
EMSE	Evidential Mean Squared Error
KL	Kullback–Leibler
OOD	Out-of-Distribution
ROC	Receiver Operating Characteristic
