# Supplementary material for: Evidential Uncertainty in Blood-Smear Image Classification: Mathematical Limits and Reliability Under Corruption
Source: Sensors (Basel). 2026 Nov 3;26(21):6957. doi: 10.3390/s26216957 (PMC13766354; doi:10.3390/s26216957)
Supplement: Supplementary file 1 [file sensors-26-06957-s001.zip › sensors-4587883-supplementary.pdf]

Supplementary Materials: Evidential Uncertainty in Blood-Smear Image Classification: Mathematical Limits and Reliability Under Corruption

Umar Hasan [1], Muhammad Ali Martuza [2]*, and Turki G. Alghamdi [3]

S1. Proofs of Propositions 1–4

This supplement provides the proofs of Propositions 1–4 in the article. The proposition numbering matches the article; equations are restated here with supplementary numbering so that all references can be resolved independently.

S1.1. Notation and Preliminaries

Let $K \geq 2$ be the number of classes, $e_k \geq 0$ the evidence for class k, and $\alpha_k = 1 + e_k$ its unit-prior Dirichlet concentration. Define

$$S = \sum_{k=1}^{K} \alpha_k, \qquad p_k = \frac{\alpha_k}{S}, \qquad u = \frac{K}{S}, \qquad b_k = \frac{\alpha_k - 1}{S}. \tag{S1}$$

Then $S \geq K$, $0 < u \leq 1$, and the subjective-opinion projection is

$$\sum_{k=1}^{K} b_k + u = 1, \qquad p_k = b_k + \frac{u}{K}. \tag{S2}$$

For a one-hot target vector y, the Evidential Mean Squared Error (EMSE) is

$$\mathcal{L}_{\text{EMSE}}(\boldsymbol{\alpha}, y) = \sum_{k=1}^{K} \left[\left(y_k - \frac{\alpha_k}{S} \right)^2 + \frac{\alpha_k (S - \alpha_k)}{S^2 (S+1)} \right]. \tag{S3}$$

S1.2. Confidence–Uncertainty Coupling

Proposition 1 (Confidence–Uncertainty Coupling). *For the unit-prior parameterization, every class probability satisfies*

$$\frac{u}{K} \leq p_k \leq 1 - \frac{K-1}{K} u. \tag{S4}$$

Consequently, if $c = \max_k p_k$ denotes top-1 confidence, then

$$\frac{1}{K} \leq c \leq 1 - \frac{K-1}{K} u, \qquad u \leq \frac{K(1-c)}{K-1}. \tag{S5}$$

Proof. Equation (S2) gives $p_k = b_k + u/K$. Because $b_k \geq 0$, the lower bound follows. Moreover, $\sum_{j \neq k} p_j \geq (K-1)u/K$, so $p_k = 1 - \sum_{j \neq k} p_j \leq 1 - (K-1)u/K$. Taking the maximum over k gives the upper bound on confidence. Since $\sum_k p_k = 1$, we also have $c \geq 1/K$. Rearranging the upper bound on c gives Equation (S5). □

S1.3. Uncertainty-Induced Brier Floor

Proposition 2 (Uncertainty-Induced Brier Floor). *Let $y = e_t$ be a one-hot target, where e_t denotes the tth standard basis vector. For any unit-prior subjective opinion with uncertainty u, the squared error of the projected mean satisfies*

$$\|y - p\|_2^2 \geq \frac{K-1}{K} u^2. \tag{S6}$$

Equality holds when all committed belief is assigned to the target class, namely $b_t = 1 - u$ and $b_j = 0$ for $j \neq t$.

Proof. For fixed u, the distance to e_t is minimized by maximizing p_t and distributing the unavoidable base-rate mass equally among non-target classes. Equation (S4) gives $p_t = 1 - (K-1)u/K$ and $p_j = u/K$ for $j \neq t$. Therefore

$$(1 - p_t)^2 + \sum_{j \neq t} p_j^2 = \left(\frac{K-1}{K}u\right)^2 + (K-1)\left(\frac{u}{K}\right)^2 = \frac{K-1}{K}u^2. \tag{S7}$$

□

S1.4. Bounds of the Evidential Mean Squared Error

Proposition 3 (Bounds of the Evidential Mean Squared Error)**.** *For $\alpha_k \geq 1$ and a one-hot target y, the EMSE satisfies*

$$0 \leq \mathcal{L}_{\text{EMSE}}(\boldsymbol{\alpha}, y) \leq 2 + \frac{K-1}{K(K+1)}. \tag{S8}$$

Proof. Both terms in Equation (S3) are nonnegative. Since $p = (p_1, \ldots, p_K)$ belongs to the probability simplex, the squared Euclidean distance between p and a simplex vertex y is at most 2. Moreover, $\sum_k p_k(1 - p_k) = 1 - \sum_k p_k^2 \leq 1 - 1/K$. Because $S \geq K$, the summed Dirichlet-variance term is bounded above by $(K-1)/[K(K+1)]$, which establishes Equation (S8). □

S1.5. Evidence-Ray Limit and Scale Degeneracy

Proposition 4 (Evidence-Ray Limit and Scale Degeneracy)**.** *Let $r \in \mathbb{R}_+^K$ with $R = \sum_k r_k > 0$, and define an evidence ray by*

$$\alpha_k(c) = 1 + c r_k, \qquad c \geq 0. \tag{S9}$$

Then

$$\begin{aligned} \lim_{c \to \infty} p_k(c) &= \frac{r_k}{R}, \qquad \lim_{c \to \infty} u(c) = 0, \\ \lim_{c \to \infty} \mathcal{L}_{\text{EMSE}}(\boldsymbol{\alpha}(c), y) &= \left\| y - \frac{r}{R} \right\|_2^2. \end{aligned} \tag{S10}$$

Proof. Along the ray, $S(c) = K + cR$ and

$$p_k(c) = \frac{1 + c r_k}{K + cR}, \qquad u(c) = \frac{K}{K + cR}. \tag{S11}$$

The stated limits for p_k and u follow by division by c. The summed variance term equals $[1 - \sum_k p_k(c)^2]/[S(c) + 1]$ and therefore converges to zero. Substitution into Equation (S3) yields Equation (S10). □
